# Hypertension as a manifestation of COVID‐19 pneumonia

**DOI:** 10.1002/ccr3.4720

**Published:** 2021-08-30

**Authors:** Makhabbat Bekbossynova, Tauekelova Ainur

**Affiliations:** ^1^ Non‐commercial joint‐stock company National Research Center for Cardiac Surgery Nur‐Sultan Kazakhstan

**Keywords:** arterial hypertension ACE2 receptors, case study, coronavirus infection, COVID‐19 pneumonia, initial manifestation, long COVID syndrome, postviral inflammatory complications

## Abstract

Several factors such as hypertension, bile duct disease, and age can affect the duration of COVID, which can lead to long COVID. Any course of coronavirus infection could have a diverse nature of clinical forms and should have a personalized approach.

## Graphical abstract

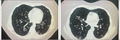

## 1 INTRODUCTION

This clinical case represents an unusual manifestation of COVID‐19 pneumonia, which started as arterial hypertension. Several factors such as hypertension, bile duct disease, and age can affect the duration of Covid. An unusual manifestation of COVID‐19 pneumonia can start with symptoms like arterial hypertension as was described in the case.

COVID‐19 has become a serious problem in the world due to its rapid spread and high mortality rate. Since the pandemic, various manifestations of COVID‐19 pneumonia have been described, ranging from classic acute respiratory viral infections to thromboembolic manifestations as the first signs of the disease.

## 2 CASE HISTORY

A 57‐year‐old female patient, a healthcare worker at the hospital, started having headache and tinnitus together with an increase in blood pressure to 160/90 mm Hg. Hypertension and other chronic illness conditions were unremarkable in the past medical history.

Previously, the patient annually underwent an in‐depth medical examination, including a study of the cardiovascular system. From the medical record, the correct sinus rhythm of the heart rate of 70 beats per minute was present on the electrocardiography; according to echocardiography, the global systolic function of the left ventricle was satisfactory, no violations of local myocardial contractility were detected, and the dimensions and cavities of the heart were not expanded. Prior to infection with SARS CoV‐2, the patient did not have an increase in blood pressure. The acute phase of coronavirus infection debuted for the first time with arterial hypertension, other catarrhal phenomena, and symptoms of infection appeared on the 3–4 days of the disease.

The patient undergoes an annual screening examination, including an in‐depth study of the cardiovascular system; no other risk factors were observed.

Elevated blood pressure values persisted for two days and were marked by ineffectiveness of antihypertensive drugs, specifically to valsartan at a dose of 80 mg two times a day. The volume of the lesion according to CT was 36% /Comment 1/.

On the third day, chills appeared, and the body temperature increased to 38.8 C.

On the fourth day, elevated blood pressure persisted without a tendency to decrease. BP was 150/90 mm Hg mm. Saturation was 96%.

**FIGURE 1:**
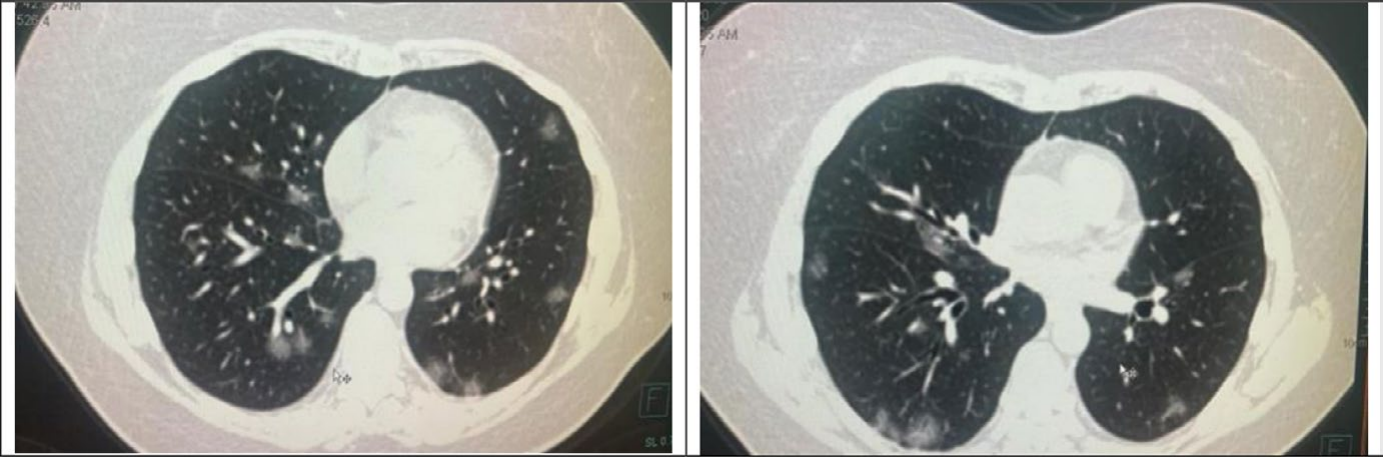
Lungs CT scan

On the fifth day, PCR for COVID‐19 was obtained, which gave a positive result, and on the lung computed tomography (CT), foci of infiltration of the pulmonary parenchyma of the "ground‐glass" type, measuring up to 5.2–2.6 cm, were found. The volume of the lesion was 22% (Figure 1).

The patient was hospitalized with a diagnosis of coronavirus infection COVID‐19 of moderate severity, PCR confirmed with bilateral polysegmental pneumonia.

### 2.1 Investigations and treatment

On laboratory assessment, CBC showed neutropenia—2.27 10^6^ L (15–61), lymphopenia—1.14 10^6^, and anesonophilia. Biochemical analysis revealed an increased level of CRP—0.51 mg/dl, ferritin—373.6 mg/L, glucose—112.58 mg/dl, increased ALT—114.59 U/L, and AST—70.13 U/L with normal bilirubin levels. It should be noted that the patient had a history of cholecystectomy (1998) and therapy for opisthorchiasis of the bile duct two years ago. Immunological study showed an increase in interleukin – 6–22.17 pg/ml.

Based on the data obtained, therapy with low molecular weight heparins was started: enoxaparin (clexane) 0.4 ml b.i.d. The antiviral drug remdesivir was prescribed according to the following scheme: day one—200 mg IV, on the following days—100 mg IV for ten days. Also, humidified oxygen was supplied nasally at a flow rate of 5 L/min for seven days, due to increased oxygen consumption noted on ABG.

On day six of the disease (the second day of hospitalization), improvement in the patient's well‐being was noted. Blood pressure reduced to 130/80 mm Hg on valsartan 160 mg per day. However, despite the ongoing therapy, body temperature up to 38 C lasted for five days and then persisted in the evening hours for up to 37.5 C for four more days. Laboratory tests showed that decrease in neutrophils ‐ 1.65 10^3^, and lymphocytes to 1.22 10^3^ and anesonophilia persisted. In the biochemical analysis, there is a slight decrease in liver function tests compared to the previous day, but the indicators remain elevated: ALT—94.8 U/L and AST—61.1 U/L. Also, an increase in CRP—1.032 mg/dl—and ferritin—484.1 µg/L—was observed.

### 2.2 Outcome and follow‐up

On the 11th day of hospitalization and the 16th day of illness, the patient's condition had stabilized, and the laboratory data continued to increase in the previous indicators: ALT 81.2 U/L, AST 38.6 U/L, CRP 2.793 mg/dl (0–0.5), and ferritin 473.1 µg/L. Normalization of neutrophils ‐ 2.52 10^3^, eosinophils ‐ 2 10^3^, but a sharp increase in platelets ‐ 533 10^9^ which could indicate the development of acute viral inflammatory syndrome. Lymphocytes in a relative amount ‐ 41%. Immunogram: interleukin 6 ‐ 29.70 pg/ml. PCR: RNA SARS‐CoV‐2 not detected (negative).

**FIGURE 2:**
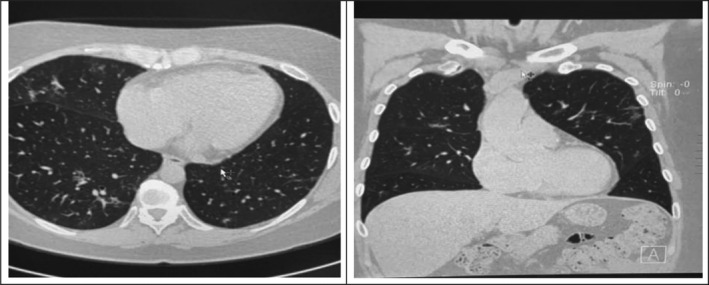
CT after a month of the disease

CT of the lungs (Figure 2) after a month of the disease showed improvement, the number of affected foci decreased, and the size of the infiltration has reduced.

## 3 DISCUSSION

This case report once again points to the link between several risk factors and prognosis in patients with COVID‐19, including hypertension, bile duct disease, and age.

Hypertension in adults is quite common, but due to the pandemic, it is of particular interest, since there are concerns that the virus uses ACE‐2 receptor to enter host cells.^1^

In this clinical case, the onset of COVID‐19 began with arterial hypertension, in the absence of risk factors for arterial hypertension. It is noteworthy that clinical manifestation in the form of high blood pressure proceeded during the latent period of COVID‐19. This is evidenced by the data of CT examination of the lungs and performed on the third day of the development of arterial hypertension. According to the CT scans, by this time, multiple ground‐glass opacities and infiltrates, which are not early signs of pneumonia, have already been found. At the same time, in the first 48 hours of the incubation period, there were no other symptoms besides arterial hypertension. This fact confirms that the detected changes in the lungs developed before or simultaneously with an increase in blood pressure. Thus, this further confirms the hypothesis that the initial stage of infection is the penetration of SARS‐CoV‐2 into target cells with ACE2 receptors. However, some experimental studies have shown that ACE2 protects against lung damage.^2^, ^3^

Special attention should be paid to the short‐term decrease in blood pressure in response to taking valsartan. At the same time, against the background of intensive treatment of pneumonia with remdesivir and ongoing hypotensive blood pressure, blood pressure returned to normal and the course of coronavirus infection did not worsen, although the effectiveness of any particular antiviral drug for treating patients with severe COVID‐19 has not been proven.^4^, ^5^

In addition, given the high transaminases—ALT, AST and normal indicators of other biochemical indicators of liver damage, such as bilirubin, alkaline phosphatase, and gamma‐glutamyltransferase in the patient, the role of the virus in liver damage remains open and requires detailed systematic study. There are studies that the virus also uses ACE2 as an entry receptor and that both liver cells and bile duct cells express ACE2. However, the expression of ACE2 in the bile duct cells is much higher than in liver cells, but is similar to the level of alveolar 2 cells type in the lungs.^6^, ^7^, ^8^

Another aspect to be explored is a genetic predisposition to an increased risk of severe SARS‐CoV‐2 infection and development of complications, which may be related to the ACE2 polymorphism that has been associated with diabetes, cerebral stroke, and hypertension, especially in Asian populations. Summarizing this information, we can say that human sensitivity may be the result of a combination of therapy and ACE2 polymorphism.^3^

Although this case has some limitations, hypertension may be taken as one of the pre‐clinical manifestation of COVID‐19, especially when it is not responding to treatment, but this cannot be generalized without further studies.

This case clearly indicates that several factors such as hypertension, bile duct disease, and age can affect the duration of COVID, which can lead to long COVID. Thus, this case clearly shows that any course of coronavirus infection could have a diverse nature of clinical forms and should have a personalized approach, taking into account risk factors and predictors, with the involvement of a multidisciplinary team to prevent long and post‐COVID‐19 syndromes.

In the context of the ongoing global pandemic, the emergence of new strains, and conflicting scientific data, clinicians have to use in daily practice in the fight against coronavirus infection treatment methods that do not currently have an evidence base, based on the clinical experience of the clinic and personal observations. To date, the presented therapy shows its effectiveness. In the future, we hope that with the accumulation of sufficient experience and knowledge, the level of evidence of these treatment methods will be determined.

## CONFLICT OF INTEREST

None.

## AUTHOR CONTRIBUTIONS

Makhabbat Bekbossynova made substantial contributions to conception and design, or acquisition of data, or analysis and interpretation of data. Tauekelova Ainur was involved in drafting the manuscript or revising it critically for important intellectual content.

## ETHICAL APPROVAL

Ethics approval and consent were given by “Local Bioethics Committee” JSC “National Research Cardiac surgery Center,” Reference number: 01‐97/2021.

## CONSENT FOR PUBLICATION

Written informed consent was obtained from the patient for publication of this case report and any accompanying images. A copy of the written consent is available for review by the editor in chief of this journal.

## Data Availability

The data that support the findings of this study are available from the corresponding author upon reasonable request. The pre‐print version of your manuscript is available online at www.researchsquare.com/article/rs‐467269/v1

## References

[1] Wan Y , Shang J , Graham R , Baric RS , Li F . Receptor recognition by the novel coronavirus from wuhan: an analysis based on decade‐long structural studies of SARS coronavirus. J Virol. 2020;94(7):e00127‐e220. doi:10.1128/JVI.00127-20 31996437 PMC7081895

[2] Zhang H , Penninger JM , Li Y , Zhong N , Slutsky AS . Angiotensin‐converting enzyme 2 (ACE2) as a SARS‐CoV‐2 receptor: molecular mechanisms and potential therapeutic target. Intensive Care Med. 2020;46(4):586‐590. 10.1007/s00134-020-05985-9 32125455 PMC7079879

[3] Fang L , Karakiulakis G , Roth M . Are patients with hypertension and diabetes mellitus at increased risk for COVID‐19 infection? Lancet Respir Med. 2020;8(4):e21. 32171062 10.1016/S2213-2600(20)30116-8 PMC7118626

[4] Norrie JD . Remdesivir for COVID‐19: challenges of underpowered studies. The Lancet. 2020;395(10236):1525–1527. 10.1016/s0140-6736(20)31023-0 PMC7190306 32423580

[5] McCreary EK , Angus DC . Efficacy of Remdesivir in COVID‐19. JAMA. 2020;324(11):1041. 10.1001/jama.2020.16337 32821934

[6] Hoffmann M , Kleine‐Weber H , Krüger N , et al. The novel coronavirus 2019 (2019‐nCoV) uses the SARS‐1 coronavirus receptor2 ACE2 and the cellular protease TMPRSS2 for entry into target cells. bioRxiv. in press. 2020. doi:10.1101/2020.01.31.929042

[7] Chai X , Hu L , Zhang Y , et al. Specific ACE2 expression in cholangiocytes may cause liver damage after 2019‐nCoV infection. bioRxiv. 2020. doi:10.1101/2020.02.03.931766

[8] Banales JM , Huebert RC , Karlsen T , Strazzabosco M , LaRusso NF , Gores GJ . Cholangiocyte pathobiology. Nat Rev Gastroenterol Hepatol. 2019;16(5):269‐281. 30850822 10.1038/s41575-019-0125-y PMC6563606

